# Identification of a Rotavirus G12 Strain, Indonesia

**DOI:** 10.3201/eid1601.091010

**Published:** 2010-01

**Authors:** Wahyu N. Wulan, Erlin Listiyaningsih, Kiki M.K. Samsi, Magdarina D. Agtini, Matthew R. Kasper, Shannon D. Putnam

**Affiliations:** US Naval Medical Research Unit No. 2, Jakarta, Indonesia (W.N. Wulan, E. Listiyaningsih, M.R. Kasper, S.D. Putnam); Sumber Waras Hospital, Jakarta (K.M.K. Samsi); Indonesian Ministry of Health, Jakarta (M.D. Agtini)

**Keywords:** G12, rotavirus, children, diarrhea, Indonesia, viruses, letter

**To the Editor:** Group A rotaviruses are the most common etiologic agents of acute gastroenteritis in infants and young children, each year resulting in ≈100 million diarrhea episodes and 600,000 deaths worldwide (*1*). The genome of rotavirus comprises 11 segments of double-stranded RNA, which encode 6 structural viral proteins (VPs) and 6 nonstructural proteins (NSPs). Recent scientific reports have identified novel rotavirus strains, such as G12 (*2**–**5*), which were first described in 1987 among Filipino children with diarrhea (*6*). In Indonesia, a rotavirus study showed that a broad variety of VP7 types (G1, G2, G3, G4, G8, G9) and VP4 types (P[4], P[6], P[8], P[9], P[10], P[11]), especially G9 and P[8] and G9P[8], were the genotype combinations most frequently encountered (*7*).

From 2005 through 2008, we conducted a nationwide surveillance study among children who had diarrhea to determine etiologies among Indonesian children seeking health services for diarrhea at hospitals and health clinics. Patients were enrolled after obtaining consent from parents/guardians of those eligible in accordance with an institutional review board protocol approved by the US Naval Medical Research Unit No. 2 (NAMRU-2) and the Ethical Committee of the Indonesian National Health Research and Development Institute. Stool specimens and clinical enrollment data were collected for each eligible patient, and all collected items were transported to NAMRU-2 in Jakarta, Indonesia. In December 2007, a stool specimen was collected from a 14-day-old afebrile infant brought to Sumber Waras Hospital in West Jakarta with diarrhea, vomiting, moderate dehydration, and malnutrition. This patient was infected with the rotavirus G12 strain, was hospitalized for 6 days, and was discharged after recovering fully. Bacterial cultures and ova/parasite evaluations were negative for enteric pathogens.

Rotavirus was detected in this specimen and genotyped by multiplex, seminested reverse transcription–PCR (RT-PCR) targeting the VP4 and VP7 genes (*8**,**9*). The specimen was typed as P[4]P[6] but was G-nontypeable. Primers to detect G12 were then used for RT-PCR and identified the proper G12 amplicon size (*2**,**3*). By use of published primers (*9*), sequencing of the VP7 gene segment confirmed the presumptive G12 genotype. Sequencing reactions were performed by using the BigDye Terminator v3.1 Cycle Sequencing Kit (Applied Biosystems, Foster City, CA, USA) on the Applied Biosystems 3130xl sequencer. Sequence analysis was done by using Sequencher 4.8 version (Gene Codes Corporation, Inc., Ann Arbor, MI, USA). Nucleotide sequences were submitted to GenBank for a BLASTN search (http://blast.ncbi.nlm.nih.gov) on the National Center for Biological Information website. We then created alignments of nucleotides and deduced amino acid sequences and compared them with a selection of reference strains from the GenBank database. Genetic relationships among G12 were determined by using PAUP version 4.0 beta 10 software (http://paup.csit.fsu.edu). A phylogenetic tree was constructed on the basis of nucleotides 1–971 of the VP7 gene by using the neighbor-joining method and applying the Kimura 2-parameter method with 1,000 bootstrap replicates of the neighbor-joining model.

The BLASTN search of the VP7 989 nucleotide sequence of the putative G12 Indonesian rotavirus (Indo SWJ0806) showed 98% similarity with published VP7 sequences of rotavirus G12 strains from Japan (CP727; GenBank accession no.AB125852), Argentina (Arg721; GenBank accession no. EU496255), and Thailand (T152; GenBank accession no. AB071404). The Indonesia G12 clustered into the lineage II composed of rotavirus G12 reference strains from Japan, Argentina, South Korea, and Thailand (Figure). Lineage II is a minority cluster when compared with lineage III, which consists of rotavirus G12 from the United States (US6588, Se585), Saudi Arabia (MD844), India (13B2), Bangladesh (RV161), and other Thailand strains (MS051) (*4*). The nucleotide sequence divergence between lineage II and lineage III ranges from 2.6% to 3.2%. Analysis of the deduced amino acid sequence alignment on the neutralization epitopes that code for the antigenic regions A, B, and C show high conservation of the most immunodominant sites (data not shown). Antigenic regions A, B, C, D, E, and F of Indonesia SWJ0806 show 100% amino acid similarity to Japan G12 strains; K12 and CP727 (*9*). The amino acid residue at position 142 of the antigenic region B has characterized lineage I and II (Val) and lineage III (Leu).

**Figure:**
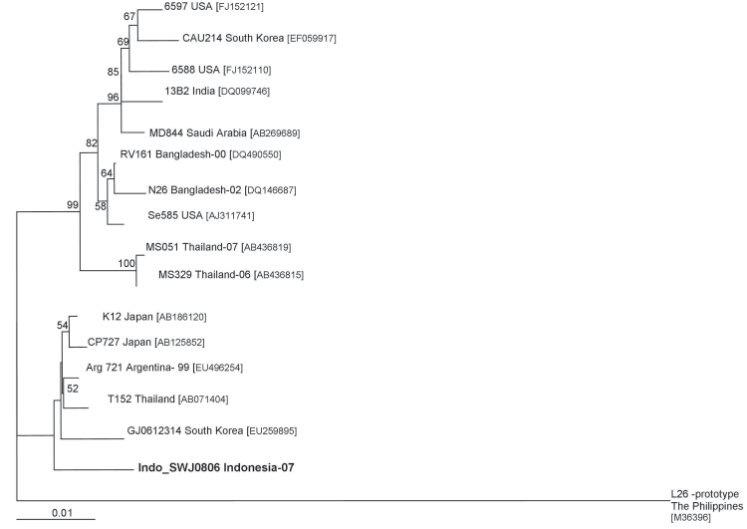
Phylogenetic analysis of the viral protein (VP) 7 genotype G12 rotavirus of Indonesia with reference strains downloaded from GenBank. The GenBank accession numbers of each strain appear next to the strain. The multiple alignment was constructed by using ClustalX version 1.81 (www.clustal.org). The phylogenetic tree was based on the 971 nt sequence of the VP7 gene and constructed by using the neighbor-joining method and applying the Kimura 2-parameter method with 1,000 bootstrap replicates of the neighbor-joining model. The isolate identified is shown in **boldface**. Bootstrap values <50% are not shown. Scale bar indicates nucleotide substitutions per site.

Phylogenetic analysis showed that the virus clusters into lineage II and that the deduced amino acid sequence is highly conserved compared with other reported rotavirus G12 strains identified. The combination of the P[6] genotype in this rotavirus strain suggests the possibility of a zoonotic transmission (*10*). Continued surveillance for rotavirus is an essential component of a country’s public health infrastructure and diarrhea prevention programs. Rotavirus genotyping from the data obtained provides necessary information for vaccine development and identification of novel and emerging rotavirus strains.
